# ANALYSIS OF 45,507 SURGICAL TREATMENT OF DISTAL TIBIA AND MALLEOLAR FRACTURES OVER 14 YEARS

**DOI:** 10.1590/1413-785220233102e263885

**Published:** 2023-05-01

**Authors:** DOV LAGUS ROSEMBERG, NELSON WOLOSKER, MARCELO FIORELLI ALEXANDRINO SILVA, TANIA SZEJNFELD MANN, VINCENZO GIORDANO, ALEXANDRE LEME GODOY-SANTOS

**Affiliations:** 1 Hospital Israelita Albert Einstein, Sao Paulo, SP, Brazil.; 2 Universidade de Sao Paulo, Faculdade de Medicina, Hospital das Clínicas, Departamento de Ortopedia e Traumatologia, Laboratório Professor Manlio Mario Marco Napoli, Sao Paulo, SP, Brazil.; 3 Universidade Federal de Sao Paulo, Escola Paulista de Medicina, Sao Paulo, SP, Brazil.; 4 Hospital Municipal Miguel Couto, Serviço de Ortopedia e Traumatologia Professor Nova Monteiro, Rio de Janeiro, RJ, Brazil.

**Keywords:** Tibial Fractures, Ankle Fractures, Fracture Dislocation, Population Characteristics, Fraturas da Tíbia, Fraturas do Tornozelo, Fratura-Luxação, Características da População

## Abstract

The distal leg joint fractures are among the most common fractures in humans across all age groups, and 50% of them require surgical treatment. Few studies discuss the epidemiology and costs of this fracture in the global and national literature. Objective: To evaluate the annual incidence and reimbursement value of distal leg joint fractures requiring surgical treatment from 2008 to 2021. Methods: A retrospective study was conducted to analyze the complex structured data of high volume and high variability (Big Data), publicly available on the TabNet platform (DATASUS), via software with artificial intelligence. Data from 2008 to 2021 on surgical treatment for malleolar fracture, distal tibia fracture, and isolated fibula fracture were analyzed. Results: From 2008 to 2021, there was an average incidence of 28.8 fractures/10^5^ inhabitants per year, representing 14.62% of all fractures. The total amount paid for hospitalizations due to these fractures was R$ 34,218,014.62 over these 14 years. Conclusion: The incidence of distal leg joint fractures follows the pattern of those recorded in other countries. The adjustment of reimbursement over the years was lower than the accumulated inflation. ***Level of Evidence II, Economic and Decision Analyses - Developing an Economic or Decision Model.***

## RESUMO

As fraturas articulares distais da perna estão entre as fraturas mais comuns do ser humano ao longo de todas as faixas etárias, e 50% delas necessitam de tratamento cirúrgico. Existem poucos trabalhos discutindo a epidemiologia e os custos dessa fratura na literatura mundial e, principalmente, na nacional. Objetivo: Avaliar a incidência anual e o valor de reembolso das fraturas distais da perna com indicação para tratamento cirúrgico entre os anos de 2008 e 2021. Métodos: Estudo retrospectivo para analisar os dados complexos estruturados de alto volume e alta variabilidade (Big Data), disponibilizados publicamente na plataforma TabNet (Datasus), através de um software com inteligência artificial. Foram analisados os dados de 2008 a 2021 do tratamento cirúrgico de fratura maleolar, fratura distal da tíbia e fratura isolada da fíbula. Resultados: Entre 2008 e 2021, houve incidência média de 28,8 fraturas/105 habitantes-ano, representando 14,62% de todas as fraturas. O valor total pago pelas internações dessas fraturas foi de R$ 34.218.014,62 ao longo desses 14 anos. Conclusão: A incidência das fraturas articulares distais da perna acompanha o padrão daqueles registrados em outros países. O reajuste do repasse ao longo dos anos foi inferior à inflação acumulada. **Nível de Evidência II, Análises Econômicas e de Decisão - Desenvolvimento de Modelo Econômico ou de Decisão**.

## INTRODUCTION

Distal joint fractures of the leg - ankle fractures (AO44) and distal fractures of the tibia (AO43) - are indicated for hospitalization for surgical treatment in up to 50% of cases,^1^ ^), (^ ^2^ which generates significant direct and indirect costs for the paying sources. ^(^ ^3^ They are common traumatic injuries in the adult population up to 60 years old, which also has a negative impact on the economically productive population. ^(^ ^4^

Moreover, epidemiological data and data related to efficiency, efficacy, values, and behavior in the production and consumption of health and health care are still scarce.

Silva et al., ^(^ ^5^ in 2020, studied the official records of hospitalization from 2004 to 2013 for the treatment of ankle fractures and reported the incidence of 21.39 fractures/10^5^ inhabitants-year in individuals over 50 years of age. American authors observed, in a population study involving all age groups, an incidence of 42.2 fractures/10^5^ inhabitants-year, ^(^ ^6^ which demonstrates a regional incidence variation for these fractures.

This study aimed to evaluate the annual incidence, length of hospital stay, and the amount paid as reimbursement of distal joint fractures of the leg with an indication for surgical treatment in the public healthcare system from 2008 to 2021.

## METHODS

This is a retrospective study that analyzes the complex structured data set of high volume and high variability (Big Data), publicly available on the TabNet platform of the public health informatics system, on one of the most populous cities in the world (DATASUS),^7^ using an artificial intelligence software.

Information regarding gender, age group, length of hospital stay, number of hospitalizations, and amount paid by the public healthcare system for the treatment of joint fractures of the leg in patients over 20 years of age were processed. The codes of the table of procedures, medications, orthoses, prostheses, and materials of the SIGTAP/SUS system were used.^8^ The following codes were used:

- 04.08.05.049-7: surgical treatment of bimalleolar/trimalleolar/ankle dislocation-fracture;
- 04.08.05.054-3: surgical treatment of tibial pilon fracture;
- 04.08.05.057-8: surgical treatment of unimalleolar ankle fracture.

For a comparative analysis, information regarding length of hospital stay, number of hospitalizations, and amount paid by the public healthcare system in the treatment of all grouped body fractures, excluding face and skull, were also processed using ICD-10 classification (codes S12, S22, S32, S42, S52, S62, S72, S82, and S92) in patients over 20 years of age. Data from the last population census of the city studied and its behavior in relation to age group and gender distribution were also extracted from the TabNet platform.^9^ ^)^ All information was collected from the website using a data collection program. It was encoded using Python v. 2.7.13 (Python Software Foundation, Beaverton, OR, USA), running on a Windows 10 operating system computer (Microsoft Corporation, Redmond, WA, USA). Data collection, field selection, and table ordering were done by the open source programs selenium-webdriver v. 3.1.8 (Selenium HQ, various developers worldwide) and pandas v. 2.7.13 (Lambda Foundry, Inc. and PyData Development Team, New York, NY, USA). After data collection, standard data transformation and cleaning procedures were performed in each file, including removing header and footer information, removing health facility code, and converting data columns into rows. The data was saved and stored in a spreadsheet in Microsoft Office Excel 2016^®^ v. 16.0.4456.1003 (Microsoft Corporation).

The data were analyzed statistically, having relevance only when p ≤ 0.05, using the ANOVA single factor and T-test method paired by mean.

## RESULTS

From 2008 to 2021, 45,507 cases of distal joint fractures of the leg were recorded in hospitals that serve the public healthcare system in the city of São Paulo - Brazil; when corrected by the total population of adults over 20 years (11,253,503), the incidence of these fractures in the population studied during the period is 28.8 fractures/10^5^ inhabitants-year on average (Table 1).

**Table 1:** Occurrence and incidence of each fracture.

	Total cases (p < 0.001)	Incidence of fractures/10^5^ inhabitants-year (p < 0.001)
**Malleolar fracture**	18,921	12.01
**Isolated fibula fracture**	22,099	14.03
**Distal tibia fracture**	4,487	2.85

**Figure 1:**
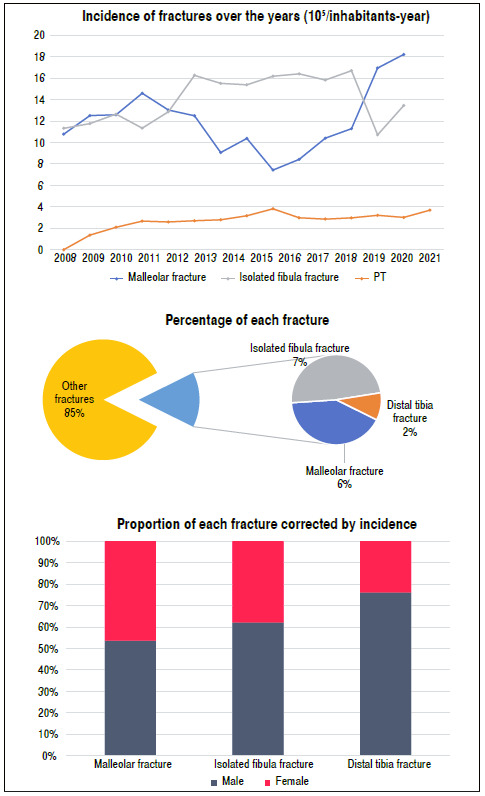
A) Incidence of each fracture over the studied years; B) Percentage of fractures in relation to total fractures over the years; C) Male/female ratio of each fracture incidence.

When we analyzed the annual pattern of these fractures, we found a statistical difference among the three fracture patterns over the years (p < 0.001), with an increase in the incidence of these fractures (Figure 1A). During the same period, 311,166 hospitalized cases of fractures of the entire skeleton, excluding face and skull, were recorded, and distal joint fractures of the leg accounted for 14.62% of the total fractures (Figure 1B).

The three types of fractures have a higher total incidence in males (p < 0.001), with distal tibia fractures presenting the highest male:female ratio of 3.2:1 and malleolar fractures presenting the lowest ratio of 1.16:1 (Figure 1C).

After age group segmentation, we noted that the three fractures occurred most commonly in the third and fourth decade of life; however, when corrected for incidence, this pattern changes according to the fracture (Table 2).

**Table 2:** Fractures incidence by age group.

Incidence	20-29	30-39	40-49	50-59	60-69	70-79	> 80
**Malleolar fracture**	15.44	16.15	17.18	19.86	20.03	13.61	7.93
**Isolated fibula fracture**	21.60	20.49	19.59	19.95	17.95	12.35	7.44
**Ankle fracture**	37.03	36.64	36.77	39.82	37.98	25.96	15.37
**Distal tibia fracture**	2.63	4.24	5.20	4.98	4.28	2.69	1.12

**Figure 2:**
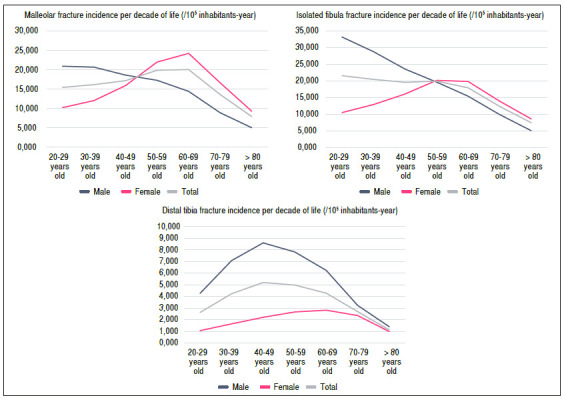
A) Malleolar fracture incidence per decade of life (/10^5^ inhabitants-year); B) Isolated fibula fracture incidence per decade of life (/10^5^ inhabitants-year); C) Distal tibia fracture incidence per decade of life (/10^5^ inhabitants-year).

When we analyzed the patients by comparing gender by age group, we found unimodal curves with peaks in different decades of the patients’ lives, with earlier peaks in man and later peaks in women. In ankle fractures, fracture prevalence showed an inversion according to the gender gender, whereas distal tibia fractures are always more prevalent in men (Figure 2).

**Figure 3:**
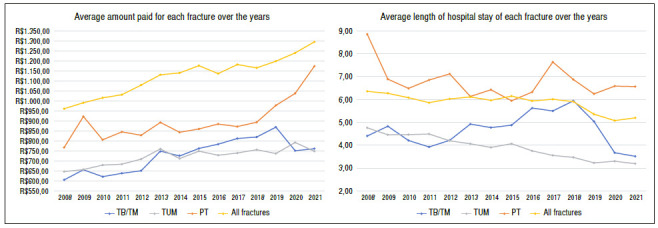
A) Average amount paid for each fracture over the years; B) Average length of hospital stay of each fracture over the years.

The total amount paid to hospitals for hospitalizations of distal joint fractures of the leg was R$ 34,218,014.62 over these 14 years. Correcting this value for the number of interactions, the average amount paid per hospitalization was R$ 783.04, ranging from R$ 672.68 in 2008 to R$ 893.81 in 2021, which is equivalent to a readjustment of 32% in 14 years (Table 3, Figure 3A).

**Table 3:** Cost per fracture.

Fracture	Average reimbursement per hospitalization (BRL)	Lowest Price (Year - R$)	Highest value (Year - R$)	Readjustment rate (%)	Reimbursement considering the average of fractures (%)
**Malleolar fracture**	728.45	2008 - 605.01	2019 - 868.45	43.54	35.18
**Isolated fibula fracture**	720.96	2008 - 646.27	2020 - 791.70	22.50	35.32
**Distal tibia fracture**	899.70	2008 - 766.77	2021 - 1,172.76	52.95	19.94
**All fractures**	1,123.77	2008 - 960.57	2021 - 1,294.44	-	34.76

When we compare the distal ankle fractures with other lower limb fractures, this discrepancy in the amount paid increases. The average value for surgical treatment of lower limb fractures was R$ 1,396.85, which represents 35.59% more than the amounts paid for the treatment of distal tibia fractures, 47.85% for malleolar fractures, and 48.39% for isolated fibula fractures.

The average length of hospital stay of distal joint fractures was 5.12 days, ranging from 6 days in 2008 to 4.42 days in 2021 (p < 0.001). When compared to the average hospital stay of the other fractures (5.25 days), distal joint fractures represent 3% less (p < 0.001) (Figure 3B).

When analyzing the codes used, we found that the code for distal tibia fractures presented a significantly longer hospital stay (6.78 days - 8.85 days in 2008 to 5.94 days in 2015), whereas the code for malleolar fractures presented an average hospital stay of 4.67 days (5.94 days in 2018 to 3.51 days in 2021) and the code for isolated fibula fracture presented an average of 3.92 days (4.76 days in 2008 to 3.19 days in 2021 - p < 0.001).

## DISCUSSION

Distal joint fractures of the leg are the most frequent cause of post-traumatic hospitalization for surgical treatment in the population up to 60 years old and represent 10.3-17% of total skeletal fractures in the adult population. ^(^ ^4^ ^), (^ ^10^ ^), (^ ^11^ In this study, distal joint fractures leg represented 14% of all fractures during the study period, whereas ankle fractures represented over 12% of all body fractures.

Somersalo et al., ^(^ ^4^ studied 6,788 cases of skeleton fractures hospitalized for treatment from 2002 to 2008. The reported ankle fracture incidence was 83 fractures/10^5^ inhabitants-year. The literature indicates a large variation in the incidence of this traumatic injury, ranging from 41.2 to 187 fractures/10^5^ inhabitants-year. ^(^ ^1^ ^), (^ ^2^ ^), (^ ^4^ ^), (^ ^6^ ^), (^ ^10^ ^)- (^ ^13^ Part of this variation in incidence is due to the fact that the studied populations present different ages and socioeconomic and cultural patterns, which reinforces the importance of using local data for strategic action in public health. Liu et al. ^(^ ^14^ found a difference in the number of cases in urban centers and rural areas.

In our study population, we observed an incidence of 28 fractures/10^5^ inhabitants-year for malleolar fractures. However, our study evaluated the number of hospitalized cases for surgical treatment, disregarding the cases that were exempted from hospitalization for non-surgical treatment. Beerekamp et al. ^(^ ^2^ reported that only 28-35% of fractures of the distal third of the leg require hospitalization for treatment and Jensen et al. ^(^ ^1^ estimated hospitalization for surgical treatment in about 50% of cases. ^(^ ^1^ ^), (^ ^2^ Applying this percentage to the studied sample, the incidence of total fractures would be approximately 52-92.85 fractures/10^5^ inhabitants-year considering only malleolar fractures, which is similar to the results found in the literature. Papin and Berthonnaud^12^ also surveyed surgical treatment fractures and found an incidence of 41.2 fractures/10^5^ inhabitants-year for malleolar fractures and 5.6 fractures/10^5^ inhabitants-year for distal tibia fractures.

In this study, isolated fractures of the fibula were 15% more frequent than malleolar fractures. However, studies have reported that malleolar fractures represent 57% of these cases in women. ^(^ ^15^ Our findings corroborate with other studies regarding less incidence of distal tibia fractures. ^(^ ^1^ ^), (^ ^15^ ^), (^ ^16^

For all types of fractures in this study, men had a higher incidence, especially in the young adult age group (peak around 20-29 years), possibly due to greater involvement in sports activities and automobile accidents, whereas women recorded more hospitalizations in older groups (peak between 60-69 years). This difference in incidence between genders observed by us differs from some findings in the literature that refer to these fractures as more common in women. ^(^ ^6^ ^), (^ ^10^ ^), (^ ^11^

Regarding the pattern of distribution throughout life, our findings corroborate most of the publications. ^(^ ^1^ ^), (^ ^2^ ^), (^ ^4^ ^), (^ ^6^ ^), (^ ^10^ ^), (^ ^13^ Scheer et al. ^(^ ^6^ also observed a higher incidence of ankle fractures in young men and women over 50 years of age.

Another point to be highlighted is that the peak incidence of isolated fibular fractures in women occurs a decade earlier than ankle fractures. The bone mineral density decreases with age in women, which can increase the risk of more complex fractures with low energy traumas. A population study in patients over 65 years of age demonstrated that ankle fractures are the second most common fracture of the lower limbs, predominantly in women. ^(^ ^5^

Studies that evaluate the risk of bone fragility observed an incidence of up to 150 fractures/10^5^ inhabitants-year in ankle fractures related to corticosteroid use, previous history of fractures, and personal history of rheumatoid arthritis. ^(^ ^16^

We observed a greater amount paid for surgical treatment for distal tibia fractures than for malleolar fractures, which is expected for more complex fractures; however, fibula isolated fractures present a greater amount paid in the SIGTAP/SUS table for hospitalization compared to malleolar fractures (bi and trimalleolar (R$481.49 and R$432.14 respectively). ^(^ ^8^ It is interesting to note how these reimbursements were established since they do not follow the degree of complexity of the surgical treatment of these fractures. The average reimbursement of distal leg fractures was lower compared to the other body fractures, and the difference is more significant when compared with other lower limb fractures. Other joint fractures, such as in the knee and hip, require more expensive implants and longer hospital stays, which may justify this difference in the amount paid.

Another point to be discussed is the price readjustment of the procedures. When we calculated the readjustment of the payment made to hospitals for the surgical treatment of these fractures, we found that the readjustment in the last 14 years was 52.95% in distal tibia fractures, 43.54% in malleolar fractures, and 22.50% in fibula isolated fractures, whereas the total readjustment of fractures treatment was 34.76%. When we used the central bank tool to calculate inflation in the same period, we found that it is significantly higher than the readjustment of reimbursement (193.741710% by the IGP-M and 124.045490% by the IPCA). ^(^ ^17^

Furthermore, when we compared the reimbursement in the Brazilian public healthcare system for these treatments to other countries, we observed a significant discrepancy in the amounts of US$ 62,000.00 in the United States (private financing system) and £ 4,730.28 in the United Kingdom (public financing system). ^(^ ^6^ ^), (^ ^18^

The value discussed in this article is the amount paid by the public health system to public hospitals, and does not represent all of the hospital costs for patients with injuries (such as surgical materials and hospital medications), which likely leads to a growing financial deficit for hospital administrations.

In this study, an average increase in the necessary hospitalization time was observed as the trauma associated with higher energy increased, and the average hospitalization time for ankle fractures, which ranged from 3.92 to 6.78, is similar to that reported by other authors. ^(^ ^5^ In a national study involving the DATA-SUS database, including 56,364 cases of ankle fractures from 2004-2013, ^(^ ^5^ ankle fractures remained, on average, 4.94 days hospitalized. Other authors report an average of 6.6 to 10.17 days of hospitalization for individuals with ankle fractures. ^(^ ^15^ ^), (^ ^18^ When we analyzed the mean length of hospitalization of SIGTAP for these fractures, we found that, on average, patients stay longer than the recommended three days for ankle fractures and four days for distal tibia fractures, ^(^ ^8^ which indicated that the reimbursement table should be adjusted.

The main limitations of this study were its retrospective nature and the inadequate completion of some data such as race, ethnicity, and educational level of the treated patients, which had to be disregarded in the data collection. We must consider that several of these hospitals are training centers for doctors, and often the documents are filled out by residents who are not properly instructed or by doctors who do not pay attention to the data. It was impossible to access information such as trauma mechanism and synthesis material used in the treatment. Despite these limitations, this is, to the best our knowledge, the first study in the Brazilian literature that analyzed the data available on the TabNet platform of the Unified Health System of the City of São Paulo (DATASUS) using an innovative robotization technology for information collection. ^(^ ^7^ This data collection strategy is faster, more efficient, and more reliable than the manual collection of each studied items, and can encourage the epidemiological study of the main diseases in the city of São Paulo, ultimately producing a high quality national literature. The estimation of the cost for the SUS with the hospital treatment of these fractures can help public administrators in budget planning and in a homogeneous reimbursement policy by municipality region and by hospitalization code.

## CONCLUSION

The incidence of distal joint fractures of the leg in the studied population was 28.8 fractures/10^5^ inhabitants-year. We noted statistically significant difference between gender and type of fracture, in which men show a younger peak incidence compared to women for the three types of fractures evaluated. The mean length of hospital stay of patients in the studied period decreased from 6 days in 2008 to 4.42 days in 2021, and showed differences according to the fracture pattern. The average amount paid by the public healthcare system (R$ 783.04) was always lower than the average of other reimbursements for fracture treatment, and the adjustment over the years was lower than the accumulated inflation.
